# Gold(I)-catalyzed formation of furans from γ-acyloxyalkynyl ketones

**DOI:** 10.3762/bjoc.9.206

**Published:** 2013-08-30

**Authors:** Marie Hoffmann, Solène Miaskiewicz, Jean-Marc Weibel, Patrick Pale, Aurélien Blanc

**Affiliations:** 1 Laboratoire de Synthèse, Réactivité Organiques et Catalyse, Institut de Chimie, UMR 7177 associé au CNRS, Université de Strasbourg, 4 rue Blaise Pascal, 67070 Strasbourg, France

**Keywords:** alkynyl ketones, cycloisomerization, furans, gold-catalysis, 1,2-migration

## Abstract

Various γ-acyloxyalkynyl ketones were efficiently converted into highly substituted furans with 2.5 mol % of triflimide (triphenylphosphine)gold(I) as a catalyst in dichloroethane at 70 °C.

## Introduction

**Scheme 1:**
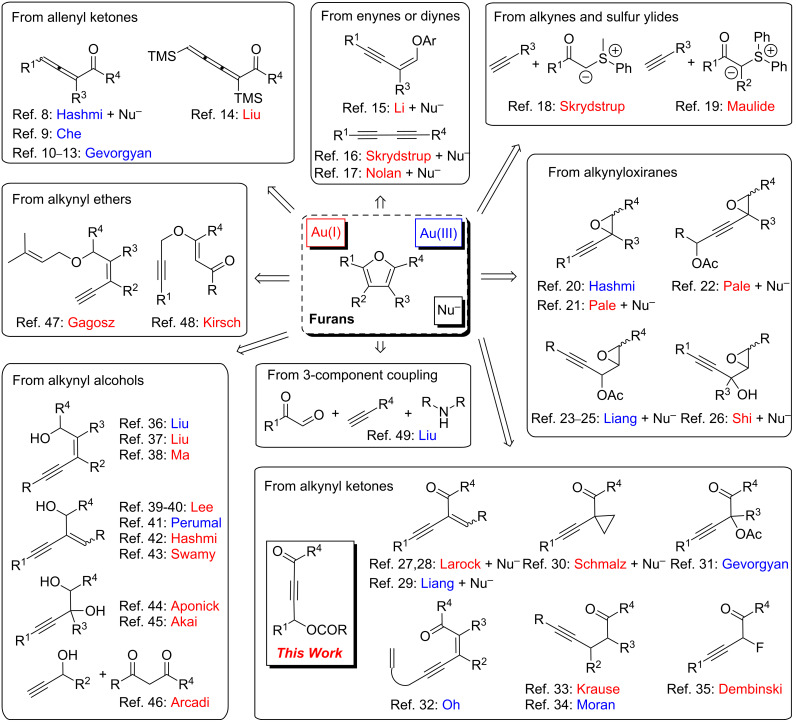
Gold(I) or gold(III)-catalyzed furan syntheses with or without nucleophiles.

Furans are an important class of aromatic compounds. They are found in many natural products, in pharmaceutical and agrochemical compounds as well as in flavor and fragrance industries [1]. Furans are also routinely used as building blocks in organic synthesis [2–3]. Therefore, a large number of synthetic methods has been developed to construct the furan motif [4–5]. Among them, late transition metal-catalyzed intra- or intermolecular cyclizations of oxygenated functionalities on unsaturated carbon–carbon bonds proved to be powerful synthetic methods due to their mildness, efficiency and diversity [6–7]. In the last decade, gold catalysts with their carbophilic character have emerged as a new tool for furan preparation. As summarized in Scheme 1, furans could now be obtained by either gold(I) or gold(III) catalysis from various types of substrates such as allenyl ketones [8–14], enynes or diynes [15–17], alkynes and sulfur ylides [18–19], alkynyl oxiranes [20–26], alkynyl ketones [27–35], alkynyl alcohols [36–46], and alkynyl ethers [47–48]. Very recently, a three-component coupling reaction toward furans catalyzed by gold(III) has been reported starting from terminal alkenes, glyoxal derivatives and secondary amines [49].

**Scheme 2:**
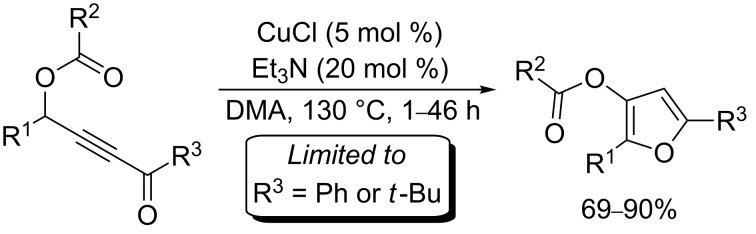
Copper(I)-catalyzed 1,2-migration/cycloisomerization of γ-acyloxyalkynyl ketones.

In this emerging research area, we have been focusing our effort on the development of furan motifs from alkynyl epoxides [21–22,50–51] and new precursors, i.e. γ-acyloxyalkynyl ketones. The latter have already been described to rearrange into furans by using copper catalysts. Indeed, Gevorgyan et al. showed that the combination of copper(I) chloride and triethylamine catalyzed the 1,2-migration/cycloisomerization of γ-acyloxyalkynyl ketones in dimethylacetamide (DMA) at 130 °C (Scheme 2) within 1–46 h [31,52–53]. Despite the relative harsh reaction conditions, furans could be obtained in good to excellent yields. However, one major limitation was ascribed to the types of the employed ketones (R^3^ in Scheme 2), as only phenyl and *tert*-butyl alkynyl ketones were able to furnish acceptable yields.

We herein report that gold(I) can overcome these limitations, providing a general, fast and very efficient transformation of γ-acyloxyalkynyl ketones into trisubstituted and functionalized furans.

## Results and Discussion

In order to find the most appropriate conditions, we applied various gold catalysts in different solvents at different temperatures to the easily available ynone **1a** (Table 1), which has been reported to afford furan **2a** in 86% yield under Gevorgyan’s conditions (Table 1, entry 1). We started our catalyst screening by using the classical combination of Ph_3_PAuCl/AgSbF_6_ and the Gagosz’s catalyst [54], i.e. (triphenylphosphine)gold(I) triflimide, in dichloroethane at room temperature. In both cases, a fast consumption of the starting material **1a** was observed compared to the copper(I) catalysis, but lower yields of furans, 44% and 65% respectively, were obtained mostly due to the formation of the hydration product **3** (Table 1, entries 2 and 3 versus entry 1). We found out that running the reaction at 70 °C instead of at room temperature completely prevented the byproduct formation. At this temperature good to quantitative yields of furans **2a** were achieved in less than 30 min, and 5 mol % of Ph_3_PAuNTf_2_ turned out to be the more efficient catalyst (Table 1, entries 2 and 3 versus entries 4 and 5). We then verified that the hydrate product was not a transient intermediate in this rearrangement by subjecting pure compound **3** to the latter reaction conditions and, even after 3 h at 70 °C, no trace of furan **2a** could be detected by ^1^H NMR analysis. Interestingly, decreasing the catalytic loading from 5 to 1 mol % still provided the furan in less than 1 h and in high yields (Table 1, entries 6 and 7 versus entry 5). However, hydration started to compete again at low loading, as evidenced by tiny amounts of **3** in the NMR spectrum of the crude (Table 1, entry 7). Control experiments revealed that other triflimide salts of coinage metals were not suited for this transformation. Indeed, silver(I) triflimide resulted mainly in degradation (Table 1, entry 8) and tetrakis(acetonitrile)copper(I) triflimide furnished the furan **2a** in only modest yield even after prolonged reaction time (Table 1, entry 9).

**Table 1:** Screening of the catalysts and the conditions.

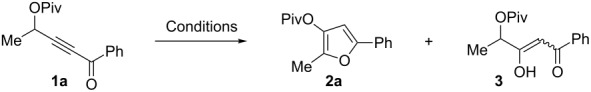						

Entry	Catalyst (mol %)	Solvent	*T* (°C)	Time (h)	Yield **2a** (%)	Yield **3** (%)

1	CuCl (5)	Et_3_N/DMA^a^	130	17	86^b^	–
2	Ph_3_PAuCl/AgSbF_6_ (5)	DCE^c^	rt	1	44	22
3	Ph_3_PAuNTf_2_ (5)	DCE	rt	2.5	65	27
4	Ph_3_PAuCl/AgSbF_6_ (5)	DCE	70	0.1	71	–
5	Ph_3_PAuNTf_2_ (5)	DCE	70	0.25	97	–
6	Ph_3_PAuNTf_2_ (2.5)	DCE	70	0.5	95	–
7	Ph_3_PAuNTf_2_ (1)	DCE	70	0.7	91	<5^d^
8	AgNTf_2_ (5)	DCE	70	16	–^e^	15^d^
9	[Cu(MeCN)_4_]NTf_2_ (5)	DCE	70	16	44	9^d^

^a^Dimethylacetamide. ^b^Reported yield from ref [52]. ^c^Dichloroethane. ^d^Estimated yield based on the ^1^H NMR of the crude mixture. ^e^Degradation occurs.

With these conditions in hand (Table 1, entry 6), we started investigating the scope of the reaction by preparing various acyloxyalkynyl ketones (Table 2). As for the phenyl alkynyl ketone **1a**, the corresponding *tert*-butyl alkynyl ketone **1b** turned out to be a good substrate for this transformation confirming Gevorgyan’s results (Table 2, entry 1 versus entry 2). Despite its bulkiness, full conversion was achieved within 30 min and 2.5 mol % of Ph_3_PAuNTf_2_, affording furan **2b** in 93% yield. We also evaluated the influence of the alkynyl substitution by increasing the size of the R^1^ group. To implement this, we introduced secondary and tertiary carbon centers next to the acyloxy function by preparing compounds **1c** (R^1^ = 2-decyl) and **1d** (R^1^ = *tert*-butyl). Compound **1c**, similar to **1a**, rearranged under these conditions, furnishing the furan **2c** in 90% yield (Table 2, entry 3). However, the presence of the sterically demanding *tert*-butyl group in **1d** drastically affected the reaction (Table 2, entry 1 and 3 versus entry 4). Even running the reaction with 5 mol % of catalyst to avoid the hydration product, the reaction took 6 h to reach almost full conversion. Beside the expected furan **2d**, its corresponding regioisomer **2d’** arising from 1,3-migration of the pivaloyl group is formed in this reaction and both products were obtained in a combined yield of 68% (Table 2, entry 4). We next varied the nature of the migratory acyloxy group. We synthetized similar substrates **1e**–**1h** bearing pivaloyl, benzoyl, acetyl and 2-phenylacetyl groups, respectively. These compounds were engaged in the gold-catalyzed process affording the furans **2e**–**2h** in the same range of yields (70–80%), suggesting that the nature of the acyloxy group had no crucial influence on the rearrangement. Indeed, the slight differences in terms of yield could be ascribed to the formation of hydration products (5–15%), and the reaction times of each reaction were inferior or equal to 1 h (Table 2, entries 5–8). We then turned our attention to the problematic R^3^ position in which only phenyl and *tert*-butyl substituents adjacent to the ketone, i.e. without enolizable position, were tolerated under copper(I)-catalyzed reaction conditions. We were pleased to observe that various other substituents, such as methyl, propyl, 2-phenylethyl, 3-benzyloxypropyl (Table 2, entries 9–12), were fully compatible with our gold-catalysis giving furans **2i**–**l** in good yields.

**Table 2:** Scope of the gold(I)-catalyzed formation of furans from γ-acyloxyalkynyl ketones.

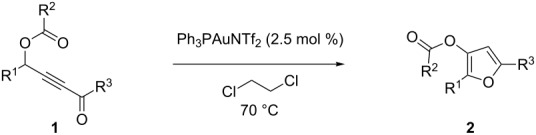				

Entry	Substrates **1**	Time (h)	Furans **2**	Yield (%)

1	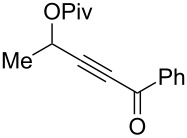 **1a**	0.5	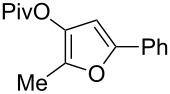 **2a**	95
2	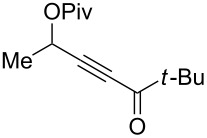 **1b**	0.5	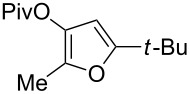 **2b**	93
3	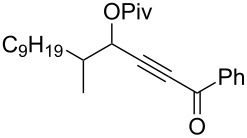 **1c**	0.75	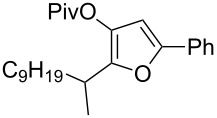 **2c**	90
4	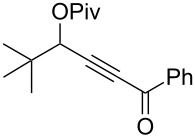 **1d**	6	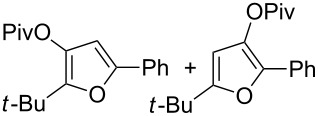 **2d**/**2d’**	68^a^
5	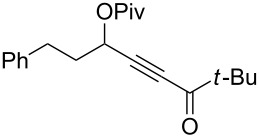 **1e**	1	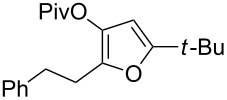 **2e**	78
6	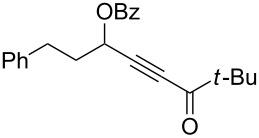 **1f**	0.5	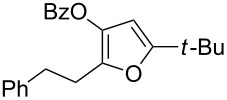 **2f**	81
7	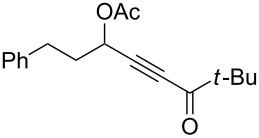 **1g**	0.75	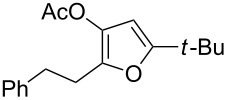 **2g**	70
8	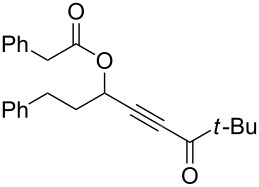 **1h**	1	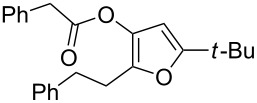 **2h**	75
9	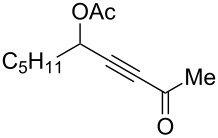 **1i**	0.33	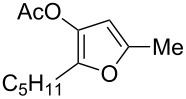 **2i**	77
10	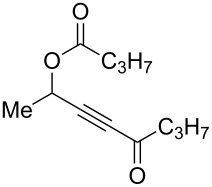 **1j**	0.5	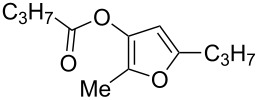 **2j**	68
11	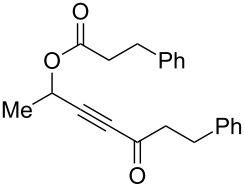 **1k**	0.33	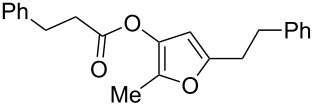 **2k**	74
12	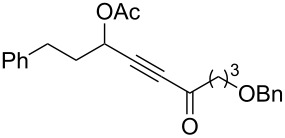 **1l**	0.33	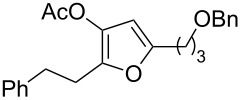 **2l**	65

^a^Cumulative yield of furans **2d** and **2d’**; reaction performed with 5 mol % of catalyst.

**Scheme 3:**
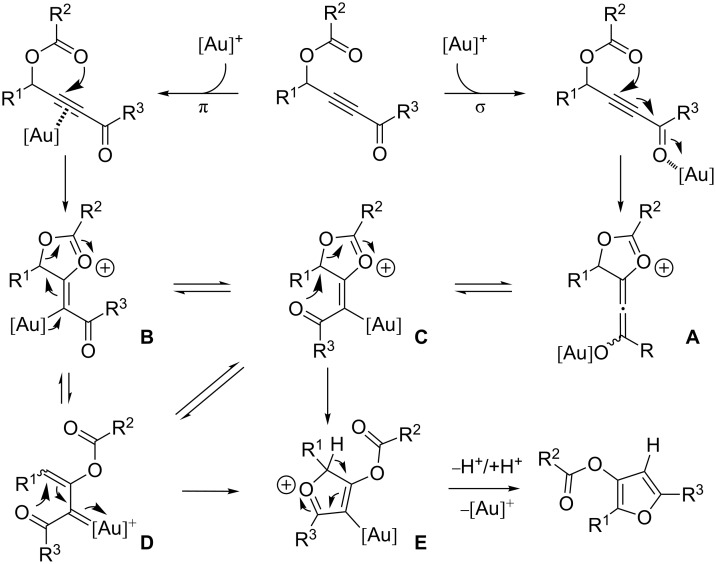
Mechanistic hypothesis for gold(I)-catalyzed conversion of γ-acyloxyalkynyl ketones into furans.

Two different mechanistic hypotheses could be envisaged in the rearrangement of γ-acyloxyalkynyl ketones into furans based on multifaceted gold-catalyst properties, i.e. the ability of gold cations to act as π or σ Lewis acids (Scheme 3) [21,55–56]. Intramolecular [1,4]-addition of the acyloxy function by oxophilic activation of γ-acyloxyalkynyl ketones could lead to the formation of gold allenolate **A**, which is in equilibrium with both *Z* or *E* vinylgold **B** and **C** [57]. Intermediate **B**, which could also be generated by carbophilic gold activation followed by nucleophilic addition of the acyloxy part, could evolve into the gold carbenoid species **D** [31]. Intermediate **C**, possessing the correct stereochemistry, and **D** might then cyclize by an attack of the carbonyl function on the carbon bearing the R^1^ substituent to afford the oxygenated five-membered ring **E**. Furan would finally be formed after tautomerization and protodemetalation of intermediate **E**.

## Conclusion

We have reported an efficient, very general and regioselective preparation of functionalized furans through a gold(I)-catalyzed rearrangement of γ-acyloxyalkynyl ketones under mild conditions. Further work is currently underway in our laboratory to fully understand this novel rearrangement.

## Experimental

**General procedure for gold(I)-catalyzed formation of furans from γ-acyloxyalkynyl ketones.** In an oven-dried flask, γ-acyloxyalkynyl ketone (0.4 mmol) was dissolved in dry dichloroethane (0.1 M) and heated to 70 °C under an argon atmosphere. Ph_3_PAuNTf_2_ (2.5 mol %) was then added to the stirred solution at 70 °C. The reaction was monitored by thin-layer chromatography until completion. The solvent was then removed in vacuo, and the crude residue was purified by silica gel flash chromatography (pentane/Et_2_O).

## Supporting Information

File 1 General procedures, characterization data and NMR spectra for compounds **1a**–**l**, **2a**–**l** and **3**.
