# Supplementary material for: Gold(I)-catalyzed formation of furans from γ-acyloxyalkynyl ketones
Source: Beilstein J Org Chem. 2013 Aug 30;9:1774–80. doi: 10.3762/bjoc.9.206 (PMC3778415; doi:10.3762/bjoc.9.206)
Supplement: File 1 — General procedures, characterization data and NMR spectra for compounds 1a–l, 2a–l and 3. [file Beilstein_J_Org_Chem-09-1774-s001.pdf]

Supporting Information

for

Gold(I)-catalyzed formation of furans from γ-acyloxyalkynyl ketones

Marie Hoffmann, Solène Miaskiewicz, Jean-Marc Weibel, Patrick Pale* and Aurélien Blanc*

Address: Laboratoire de Synthèse, Réactivité Organiques et Catalyse, Institut de Chimie, UMR 7177 associé au CNRS, Université de Strasbourg, 4 rue Blaise Pascal, 67070 Strasbourg, France

Email: Patrick Pale* - ppale@unistra.fr and Aurélien Blanc* - ablanc@unistra.fr

*Corresponding author

General procedures, characterization data and NMR spectra for compounds 1a–l, 2a–l and 3

Contents

General Information S2

Preparation and characterization of compounds S2

Proton and Carbon NMR spectra for all compounds S9

General Information:

Proton (^{1}H NMR) and carbon (^{13}C NMR) nuclear magnetic resonance spectra were recorded on 300, 400 or 500 MHz instruments. The chemical shifts are given in part per million (ppm) on the delta scale. The solvent peak was used as reference value. For ^{1}H NMR: $CDCl_3$ = 7.26 ppm. For ^{13}C NMR: $CDCl_3$ = 77.16 ppm. Data are presented as follow: chemical shift, multiplicity (s = singlet, d = doublet, t = triplet, q = quartet, quint = quintet, m = multiplet), coupling constants (*J*/Hz) and integration. Assignments were determined either on the basis of unambiguous chemical shifts or coupling patterns, COSY, HMQC, HMBC, NOESY experiments to fully interpreted spectra for related compounds. Infrared spectra were recorded neat with Hewlett-Packard spectrometer. Wavelengths of maximum absorbance (ν_{max}) are quoted in wave numbers (cm^{-1}). High resolution mass spectra (HRMS) data were recorded on a microTOF spectrometer equipped with orthogonal electrospray interface (ESI). The parent ions $[M]^+$, $[M + H]^+$, $[M + Li]^+$ or $[M + Na]^+$ are quoted. Analytical thin-layer chromatography (TLC) was carried out on silica gel 60 F254 plates or basic alumina (63−200 μm) with visualization by ultraviolet light, cerium ammonium molybdate (CAM) or potassium permanganate dip. Flash-column chromatography was carried out using silica gel 60 (40−63 μm) or basic Al_2O_3 (63−200 μm) and the procedure included the subsequent evaporation of solvents in vacuo. Reagents and solvents were purified using standard means. Dichloroethane ($(CH_2Cl)_2$), and triethylamine (NEt_3) were distilled from CaH_2 under an argon atmosphere; pyridine was distilled from potassium carbonate; diisopropylamine (DIPA) was distilled from KOH; and tetrahydrofuran (THF) was dried using Glasstechnology GT S100 device. Anhydrous reactions were carried out in flame-dried glassware and under an argon atmosphere.

CuI (98%) and DMA (≥99.5%) was purchased from Sigma Aldrich. $AgSbF_6$ (98%) was purchased from STREM Chemicals. $AgNTf_2$ was prepared from commercially available $HNTf_2$ (Aldrich) and Ag_2CO_3 [1]. Triphenylphosphinegold(I) chloride was prepared by reduction of $NaAuCl_4$ with thiodiethanol and subsequent addition of triphenylphosphine [2]. PPh_3AuNTf_2 catalyst was prepared from the corresponding triphenylphosphinegold(I) chloride and $AgNTf_2$ [3]. $Cu(MeCN)_4NTf_2$ was prepared from commercially available $HNTf_2$ (Aldrich) and Cu_2O (Acros) [4]. All other chemicals were used as received. All extractive procedures were performed using non distilled solvents and all aqueous solutions used were saturated unless details are given.

Procedure A: preparation of γ-acyloxyalkynyl ketone by Sonogashira reaction [5]

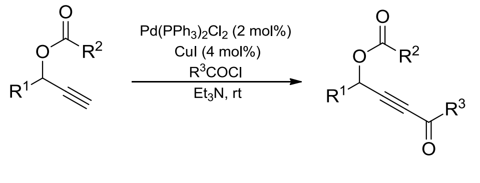

To an oven-dried flask under argon were successively added copper iodide (4 mol %), dichlorobis(triphenylphosphine)palladium (2 mol %) and NEt_3 [0.4 M]. The acyloxyalkyne (1 equiv) previously dissolved in NEt_3 [0.5 M] was added to the catalyst mixture via cannula

[1] Vij, A.; Zheng, Y. Y.; Kirchmeier, R. L.; Shreeve, J. M. *Inorg. Chem.* **1994**, *33*, 3281−3288.
[2] Al'Sa-Ady, A. K.; McAuliffe, C. A.; Parish, R. V.; Sandeank, J. A. *Inorg. Synth.* **1985**, 191−194.
[3] Mezailles, N.; Ricard, L.; Gagosz, F. *Org. Lett.* **2005**, *7*, 4133−4136.
[4] Gronnier C.; Kramer S.; Odabachian Y.; Gagosz F. *J. Am. Chem. Soc.* **2012**, *134*, 828–831.
[5] Schwier, T.; Sromek, A. W.; Yap, D. M. L.; Chernyak, D.; Gevorgyan, V. *J. Am. Chem. Soc.* **2007**, *129*, 9868−9878.

and briefly stirred. The acid chloride (1.1 equiv) was then added dropwise. The reaction was stirred at room temperature and monitored by TLC until completion. The mixture was quenched with HCl (1 N) and extracted with Et_2O. The organic layers were washed with HCl (1 N), $NaHCO_3$ solution and brine, dried over $MgSO_4$ and concentrated under vacuum. The resulting crude product was purified by flash-column chromatography on silica gel (cyclohexane/EtOAc).

Procedure B: preparation of γ-acyloxyalkynyl ketone by condensation [5]

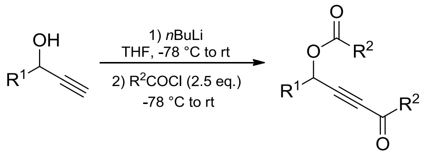

To a stirred solution of propargyl alcohol (1 equiv) in THF [0.5 M] under argon at −78 °C, was dropwise added *n*-BuLi ([1.6 M] in hexanes, 2.2 equiv). The resulting solution was then allowed to warm to room temperature and stirred for 0.5 to 1 hour. A solution of acid chloride (2.5 equiv) in THF [1.8 M] under argon at −78 °C was added via cannula to the mixture. The reaction was then allowed to warm to room temperature and stirred for 2 to 7 hours (monitored by TLC). The reaction was quenched with NH_3 (aqueous solution) and extracted with Et_2O. The organic layers were washed with HCl (1 N), $NaHCO_3$ and brine, dried over $MgSO_4$ and concentrated under vacuum. The resulting crude product was purified by column chromatography on silica gel (cyclohexane/EtOAc).

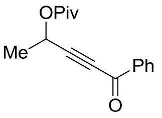

5-Oxo-5-phenylpent-3-yn-2-yl pivalate (1a) [5]: Prepared following the **Procedure A** in 63% yield (1.06 g, 4.10 mmol) from 1.00 g (6.48 mmol) of but-3-yn-2-yl pivalate. Colorless oil; TLC R_f 0.60 (cyclohexane/EtOAc 25%); ^{1}H NMR (400 MHz, $CDCl_3$) δ 1.26 (s, 9H), 1.63 (d, J = 6.8 Hz, 3H), 5.66 (q, J = 6.8 Hz, 1H), 7.48 (dd, J = 8.1, 7.5 Hz, 2H), 7.62 (t, J = 7.5 Hz, 1H), 8.10 (d, J = 8.1 Hz, 2H); ^{13}C NMR (125.8 MHz, $CDCl_3$) δ 20.5, 27.2, 38.9, 59.8, 82.0, 92.0, 128.7, 129.7, 134.4, 136.6, 177.3, 177.6.

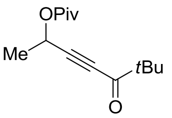

6,6-Dimethyl-5-oxohept-3-yn-2-yl pivalate (1b): Prepared following the **Procedure B** in 38% yield (1.30 g, 5.45 mmol) from 1.00 g (14.30 mmol) of but-3-yn-2-yl pivalate. Colorless oil; TLC R_f 0.40 (pentane/Et_2O 5%); IR (neat) ν_{max} 1007, 1035, 1079, 1133, 1274, 1366, 1396, 1460, 1479, 1675, 1736, 2217, 2872, 2936, 2971 cm^{-1}; ^{1}H NMR (300 MHz, $CDCl_3$) δ 1.18 (s, 9H), 1.22 (s, 9H), 1.55 (d, J = 6.8 Hz, 3H), 5.55 (q, J = 6.8 Hz, 1H); ^{13}C NMR (125.8 MHz, $CDCl_3$) δ 20.4, 26.0, 27.1, 38.9, 45.0, 59.7, 81.3, 91.4, 177.2, 193.7; HRMS 261.148 ($C_{14}H_{21}O_3$ + Na calcd 261.146).

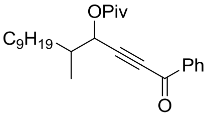

5-Methyl-1-oxo-1-phenyltetradec-2-yn-4-yl pivalate (1c): Prepared following the **Procedure A** in 56% yield (229 mg, 0.57 mmol, mixture 50/50 of 2 diastereoisomers) from 300 mg (1.02 mmol) of 4-methyltridec-1-yn-3-yl pivalate. Colorless oil; TLC R_f 0.56 (cyclohexane/EtOAc 10%); IR (neat) ν_{max} 977, 1031, 1133, 1259, 1312, 1365, 1396, 1450, 1582, 1598, 1650, 1736, 2208, 2226, 2854, 2924, 2958, 3067 cm^{-1}; ^{1}H NMR (300 MHz, $CDCl_3$) δ 0.83–0.92 (m, 6H), 1.10 (d, J = 6.9 Hz, 3H, Diastereoisomer 1), 1.11 (d, J = 6.9 Hz, 3H, Diastereoisomer 2), 1.21–1.33 (m, 28H), 1.26–1.27 (m, 18H), 1.35–1.45 (m, 2H), 1.52–1.64 (m, 2H), 1.95–2.05 (m, 2H), 5.50 (d, J = 5.0 Hz, 1H, Diastereoisomer 2), 5.52 (d, J = 5.4 Hz, 1H, Diastereoisomer 1), 7.50–7.45 (m, 4H), 7.59–7.64 (m, 2H), 8.08–8.12 (m, 4H); ^{13}C NMR (125.8 MHz, $CDCl_3$) δ

14.27 (x2), 15.3, 15.6, 22.8 (x2), 27.0, 27.1, 27.2 (x2), 29.5 (x2), 29.6 (x2), 29.7 (x2), 29.8, 29.9, 32.0 (x2), 32.4, 32.7, 37.2, 37.5, 39.1, 39.2, 67.4, 67.8, 83.2, 83.5, 90.5, 91.1, 128.7 (x2), 129.7 (x2), 134.4 (x2), 136.7 (x2), 177.3, 177.4, 177.50, 177.53; HRMS 421.270 ($C_{26}H_{38}O_3$ + Na calcd 421.271).

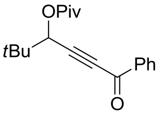

2,2-Dimethyl-6-oxo-6-phenylhex-4-yn-3-yl pivalate (1d): Prepared following the **Procedure A** in 70% yield (1.30 g, 4.33 mmol) from 1.20 g (6.12 mmol) of 4,4-dimethylpent-1-yn-3-yl pivalate. White solid; mp 54 °C; TLC R_f 0.40 (cyclohexane/EtOAc 10%); IR (neat) ν_{max} 1110, 1266, 1313, 1366, 1395, 1448, 1478, 1580, 1596, 1638, 1739, 2238, 2871, 2935, 2972, 3071 cm^{-1}; ^{1}H NMR (500 MHz, $CDCl_3$) δ 1.11 (s, 9H), 1.28 (s, 9H), 5.30 (s, 1H), 7.48 (dd, J = 7.5, 7.3 Hz, 2H), 7.61 (t, J = 7.2 Hz, 1H), 8.10 (d, J = 7.5 Hz, 2H); ^{13}C NMR (125.8 MHz, $CDCl_3$) δ 25.9, 27.2, 35.9, 39.2, 71.5, 83.3, 90.6, 128.8, 129.7, 134.4, 136.8, 177.3, 177.5; HRMS 323.163 ($C_{19}H_{24}O_3$ + Na calcd 323.162).

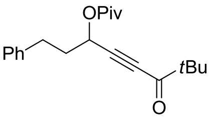

7,7-Dimethyl-6-oxo-1-phenyloct-4-yn-3-yl pivalate (1e): Prepared following the **Procedure A** in 84% yield (553 mg, 1.68 mmol) from 489 mg (2.00 mmol) of 5-phenylpent-1-yn-3-yl pivalate. Colorless oil; TLC R_f 0.50 (cyclohexane/CH_2Cl_2 50%); IR (neat) ν_{max} 699, 745, 1032, 1130, 1275, 1365, 1478, 1496, 1674, 1736, 2214, 2870, 2933, 2969, 3029 cm^{-1}; ^{1}H NMR (500 MHz, $CDCl_3$) δ 1.19 (s, 9H), 1.24 (s, 9H), 2.11−2.23 (m, 2H), 2.79 (t, J = 8.0 Hz, 2H), 5.74 (t, J = 6.8 Hz, 1H), 7.18 (d, J = 7.7 Hz, 2H), 7.21 (t, J = 7.2 Hz, 1H), 7.30 (dd, J = 7.7, 7.2 Hz, 2H); ^{13}C NMR (125.8 MHz, $CDCl_3$) δ 26.0, 27.2, 31.3, 35.7, 39.0, 45.0, 62.8, 82.2, 90.3, 126.5, 128.5, 128.8, 140.3, 177.2, 193.7; HRMS 351.193 ($C_{21}H_{28}O_3$ + Na calcd 351,194).

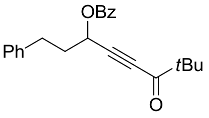

7,7-Dimethyl-6-oxo-1-phenyloct-4-yn-3-yl benzoate (1f): Prepared following the **Procedure A** in 70% yield (478 mg, 1.37 mmol) from 518 mg (1.96 mmol) of 5-phenylpent-1-yn-3-yl benzoate. Colorless oil; TLC R_f 0.23 (pentane/Et_2O 5%); IR (neat) ν_{max} 711, 745, 1025, 1067, 1094, 1141, 1261, 1476, 1672, 1723, 2214, 2868, 2931, 2968, 3028 cm^{-1}; ^{1}H NMR (300 MHz, $CDCl_3$) δ 1.21 (s, 9H), 2.23−2.42 (m, 2H), 2.89 (t, J = 7.9 Hz, 2H), 5.74 (t, J = 6.6 Hz, 1H), 7.18−7.25 (m, 3H), 7.27−7.34 (m, 2H), 7.47 (dd, J = 8.3, 7.5 Hz, 2H), 7.60 (t, J = 7.5 Hz, 1H), 8.04 (d, J = 8.3 Hz, 2H); ^{13}C NMR (125.8 MHz, $CDCl_3$) δ 26.0, 31.5, 35.9, 45.0, 63.5, 82.6, 90.0, 126.5, 128.5, 128.6, 128.8, 129.4, 129.9, 133.6, 140.2, 165.4, 193.6; HRMS 371.158 ($C_{23}H_{24}O_3$ + Na calcd 371.163).

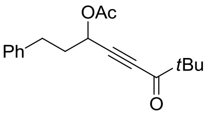

7,7-Dimethyl-6-oxo-1-phenyloct-4-yn-3-yl acetate (1g): Prepared following the **Procedure A** in 57% yield (331 mg, 1.16 mmol) from 410 mg (2.03 mmol) of 5-phenylpent-1-yn-3-yl acetate. Colorless oil; TLC R_f 0.28 (pentane/Et_2O 10%); IR (neat) ν_{max} 699, 745, 1023, 1222, 1369, 1476, 1673, 1745, 2213, 2869, 2933, 2969, 3028 cm^{-1}; ^{1}H NMR (500 MHz, $CDCl_3$) δ 1.20 (s, 9H), 2.10 (s, 3H), 2.11−2.23 (m, 2H), 2.79 (t, J = 7.9 Hz, 2H), 5.47 (t, J = 6.7 Hz, 1H), 7.18 (d, J = 7.8 Hz, 2H), 7.21 (t, J = 7.5 Hz, 1H), 7.30 (dd, J = 7.8, 7.5 Hz, 2H); ^{13}C NMR (125.8 MHz, $CDCl_3$) δ 21.0, 26.0, 31.4, 35.7, 45.0, 63.0, 82.4, 89.9, 126.5, 128.5, 128.8, 140.23, 169.8, 193.6; HRMS 309.149 ($C_{18}H_{22}O_3$ + Na calcd 309.147).

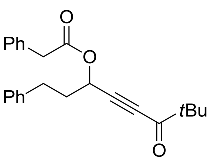

7,7-Dimethyl-6-oxo-1-phenyloct-4-yn-3-yl 2-phenylacetate (1h): Prepared following the **Procedure A** in 66% yield (204 mg, 0.56 mmol) from 489 mg (0.86 mmol) of 5-phenylpent-1-yn-3-yl 2-phenylacetate. Colorless oil; TLC R_f 0.27 (pentane/Et_2O 15%); IR (neat) ν_{max} 1131, 1240, 1455, 1476, 1496, 1672, 2213, 2868, 2931, 2968, 3029, 3063, 3087 cm^{-1}; ^1H NMR (300 MHz, $CDCl_3$) δ 1.54 (s, 9H), 2.06–2.24 (m, 2H), 2.71 (t, J = 7.8 Hz, 2H), 3.65 (s, 2H), 5.47 (t, J = 6.6 Hz, 1H), 7.10 (d, J = 7.6 Hz, 2H), 7.20 (t, J = 7.3 Hz, 1H), 7.25–7.37 (m, 7H); ^{13}C NMR (125.8 MHz, $CDCl_3$) δ 26.0, 31.2, 35.7, 41.3, 45.0, 63.3, 82.5, 89.7, 126.5, 127.5, 128.5, 128.7, 128.8, 129.4, 133.5, 140.2, 170.3, 193.6; HRMS 385.176 ($C_{24}H_{26}O_3$ + Na calcd 385.177).

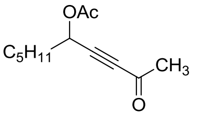

2-Oxodec-3-yn-5-yl acetate (1i) [6]: Prepared following the **Procedure B** in 52% yield (2.11 g 10.03 mmol) from 2.8 mL (19.68 mmol) of oct-1-yn-3-ol. Colorless oil; TLC R_f 0.50 (cyclohexane/EtOAc 20%); ^1H NMR (300 MHz, $CDCl_3$) δ 0.90 (t, J = 6.9 Hz, 3H), 1.27−1.37 (m, 4H), 1.38−1.50 (m, 2H), 1.76−1.86 (m, 2H), 2.10 (s, 3H), 2.35 (s, 3H), 5.46 (t, J = 6.7 Hz, 1H); ^{13}C NMR (75.5 MHz, $CDCl_3$) δ 14.1, 21.0, 22.6, 24.7, 31.3, 32.7, 34.1, 63.4, 84.3, 88.5, 169.9, 184.

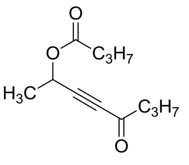

5-Oxooct-3-yn-2-yl butyrate (1j): Prepared following the **Procedure B** in 58% yield (876 mg, 4.17 mmol) from 0.5 mL (7.13 mmol) of but-3-yn-2-ol. Colorless oil; TLC R_f 0.38 (pentane/Et_2O 20%); IR (neat) ν_{max} 1023, 1078, 1159, 1245, 1350, 1456, 1678, 1742, 2216, 2876, 2937, 2965 cm^{-1}; ^1H NMR (300 MHz, $CDCl_3$) δ 0.93 (t, J = 7.4 Hz, 3H), 0.96 (t, J = 7.4 Hz, 3H), 1.53 (d, J = 6.8 Hz, 3H), 1.67 (qt, J = 7.4, 7.4 Hz, 2H), 1.68 (qt, J = 7.4, 7.4 Hz, 2H), 2.32 (t, J = 7.4 Hz, 2H), 2.53 (t, J = 7.4 Hz, 2H), 5.56 (q, J = 6.8 Hz, 1H); ^{13}C NMR (125.8 MHz, $CDCl_3$) δ 13.6, 13.7, 17.5, 18.5, 20.6, 36.1, 47.3, 59.4, 83.1, 89.3, 172.4, 187.6; HRMS 233.116 ($C_{12}H_{18}O_3$ + Na calcd 233.115).

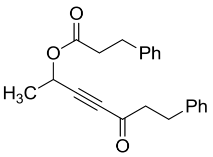

5-Oxo-7-phenylhept-3-yn-2-yl 3-phenylpropanoate (1k): Prepared following the **Procedure B** in 45% yield (1.89 g, 5.69 mmol) from 1 mL (12.76 mmol) of but-3-yn-2-ol. Yellowish oil; TLC R_f 0.31 (pentane/Et_2O 20%); IR (neat) ν_{max} 698, 748, 1030, 1076, 1087, 1138, 1229, 1453, 1496, 1678, 1740, 2220, 2934, 3028 cm^{-1}; ^1H NMR (300 MHz, $CDCl_3$) δ 1.50 (d, J = 6.9 Hz, 3H), 2.67 (t, J = 7.6 Hz, 2H), 2.89 (t, J = 7.6 Hz, 2H), 2.97 (m, 4H), 5.54 (q, J = 6.9 Hz, 1H), 7.16−7.23 (m, 6H), 7.26−7.32 (m, 4H); ^{13}C NMR (125.8 MHz, $CDCl_3$) δ 20.5, 29.7, 30.9, 35.8, 47.0, 59.7, 83.1, 89.6, 126.5, 126.5, 128.4, 128.5, 128.7, 128.7, 140.1, 140.2, 171.6, 186.3; HRMS 357,144 ($C_{22}H_{22}O_3$ + Na calcd 357,146).

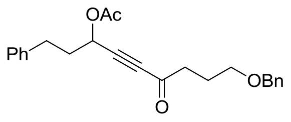

9-(Benzyloxy)-6-oxo-1-phenylnon-4-yn-3-yl acetate (1l): To a stirred solution of 1-(benzyloxy)-7-hydroxy-9-phenylnon-5-yn-4-one [7] (500 mg, 1.31 mmol) in dichloromethane was added at once a mixture of pyridinium chlorochromate (430 mg, 2 mmol) and SiO_2 (430 mg). After 5 h of stirring at room temperature, the reaction mixture was filtered through a pad of Celite® (CH_2Cl_2) and the filtrate was concentrated in vacuo. The crude product was purified by flash chromatography (cyclohexane/EtOAc 10-20%) to afford **1l** as a pale yellow oil (460 mg, 1.22 mmol, 93%). TLC R_f 0.45 (cyclohexane/EtOAc 25%); IR (neat) ν_{max} 698, 737, 1025, 1101, 1155, 1220, 1369, 1454, 1496, 1677, 1743, 2217, 2859, 2931, 3028 cm^{-1}; ^1H NMR (300 MHz, $CDCl_3$) δ 1.97 (tt, J = 6.0, 7.3 Hz, 2H), 2.09 (s, 3H), 2.10–2.19 (m, 2H),

[6] Sromek, A. W.; Kel'in, A. V.; Gevorgyan, V. *Angew. Chem., Int. Ed.* **2004**, *43,* 2280–2282.
[7] This compound was synthetized according to the sequence described by Singh, M.; Argade, N. P. *J. Org. Chem.* **2010**, *75*, 3121–3124.

2.71 (t, J = 7.3 Hz, 2H), 2.78 (t, J = 7.9 Hz, 2H), 3.50 (t, J = 6.1 Hz, 2H), 4.49 (s, 2H), 5.45 (t, J = 6.67 Hz, 1H), 7.16-7.34 (m, 10H); ^{13}C NMR (125.8 MHz, $CDCl_3$) δ 20.9, 24.0, 31.3, 35.6, 42.4, 62.9, 69.0, 73.0, 84.1, 88.2, 126.5, 127.7, 128.5, 128.5, 128.7, 138.4, 140.2, 169.8, 186.9; HRMS 401.174 ($C_{24}H_{26}O_4$ + Na calcd 401.172).

Procedure C: gold(I)-catalyzed formation of furans from γ-acyloxyalkynyl ketones

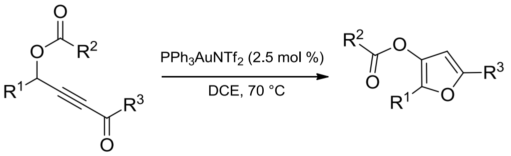

In an oven-dried flask, γ-acyloxyalkynyl ketone (0.4 mmol) was dissolved in dichloroethane [0.1 M] and heated to 70 °C under an argon atmosphere. $Ph_3PAuNTf_2$ (2.5 mol %) was then added to the stirred solution at 70 °C. The reaction was monitored by thin-layer chromatography until completion. The solvent was then removed in vacuo and the crude residue was purified by flash chromatography on silica gel (pentane/Et_2O).

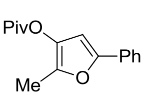

2-Methyl-5-phenylfuran-3-yl pivalate (2a) [5]: Prepared following the **Procedure C** in 95% yield (99 mg, 0.38 mmol) from 102 mg (0.40 mmol) of **1a**. White solid; mp 57 °C; TLC R_f 0.60 (Pentane/Et_2O 5%); ^{1}H NMR (400 MHz, $CDCl_3$) δ 1.35 (s, 9H), 2.26 (s, 3H), 6.64 (s, 1H), 7.23 (t, J = 7.3 Hz, 1H), 7.36 (dd, J = 7.9, 7.3 Hz, 2H), 7.59 (d, J = 7.8 Hz, 2H); ^{13}C NMR (125.8 MHz, $CDCl_3$) δ 10.8, 27.3, 39.2, 102.1, 123.4, 127.3, 128.7, 131.0, 135.9, 139.8, 150.1, 176.2.

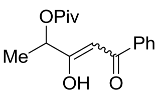

3-Hydroxy-5-oxo-5-phenylpent-3-en-2-yl pivalate (3): By-product obtained following the **Procedure C** (at room temperature instead of 70 °C) in 22% yield (22 mg, 0.09 mmol) from 101 mg (0.39 mmol) of **1a**. Yellowish oil; TLC R_f 0.31 (pentane/Et_2O 5%); IR (neat) ν_{max} 521, 633, 685, 761, 879, 1036, 1060, 1116, 1150, 1278, 1477, 1569, 1598, 1731, 2903, 2958 cm^{-1}; ^{1}H NMR (300 MHz, $CDCl_3$) δ 1.30 (s, 9H), 1.50 (d, J = 7.0 Hz, 3H), 5.28 (q, J = 7.0 Hz, 1H), 6.30 (s, 1H), 7.46 (dd, J = 7.5, 7.1 Hz, 2H), 7.54 (t, J = 7.1 Hz, 1H), 7.86 (d, J = 7.5 Hz, 2H), 15.79 (s, 1H); ^{13}C NMR (125.8 MHz, $CDCl_3$) δ 17.9, 27.3, 38.9, 71.9, 92.4, 127.1, 128.9, 132.7, 134.4, 177.6, 182.6, 196.1. HRMS 276.137 ($C_{16}H_{20}O_4$ + Na calcd 276.136).

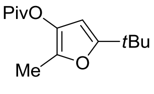

5-(*tert*-Butyl)-2-methylfuran-3-yl pivalate (2b): Prepared following the **Procedure C** in 93% yield (101 mg, 0.42 mmol) from 108 mg (0.45 mmol) of **1b**. Colorless oil; TLC R_f 0.77 (pentane/Et_2O 5%); IR (neat) ν_{max} 939, 1028, 1117, 1190, 1241, 1277, 1363, 1395, 1460, 1479, 1652, 1735, 2871, 2914, 2967 cm^{-1}; ^{1}H NMR (300 MHz, $CDCl_3$) δ 1.23 (s, 9H), 1.31 (s, 9H), 2.14 (s, 3H), 5.95 (s, 1H); ^{13}C NMR (125.8 MHz, $CDCl_3$) δ 10.7, 27.3, 29.0, 32.8, 39.1, 99.3, 134.2, 137.6, 160.5, 176.5; HRMS 261.146 ($C_{14}H_{22}O_3$ + Na calcd 261.146).

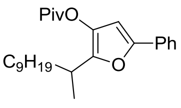

5-Phenyl-2-(undecan-2-yl)furan-3-yl pivalate (2c): Prepared following the **Procedure C** in 90% yield (45 mg, 0.11 mmol) from 49 mg (0.12 mmol) of **1c**. Colorless oil; TLC R_f 0.38 (pentane/Et_2O 5%); IR (neat) ν_{max} 1113, 1175, 1275, 1397, 1456, 1604, 1754, 2838, 2924, 2971 cm^{-1}; ^{1}H NMR (300 MHz, $CDCl_3$) δ 0.86 (t, J = 7.0 Hz, 3H), 1.20–1.27 (m, 14H), 1.29 (d, J = 7.2 Hz, 3H), 1.34 (s, 9H), 1.51–1.59 (m, 1H), 1.65–1.74 (m, 1H), 2.83 (dqd, J = 8.4, 7.2, 5.8 Hz, 1H), 6.65 (s, 1H), 7.22 (t, J = 7.4 Hz, 1H), 7.35 (dd, J = 8.4, 7.4 Hz, 2H), 7.59 (d, J = 8.4 Hz, 2H); ^{13}C NMR (125.8 MHz, $CDCl_3$) δ

14.3, 19.0, 22.8, 27.3, 27.6, 29.5, 29.6, 29.7, 29.7, 31.3, 32.0, 35.6, 39.2, 102.1, 123.4, 127.2, 128.7, 131.1, 135.0, 146.9, 149.6, 176.3; HRMS 421.272 ($C_{26}H_{38}O_3$ + Na calcd 421.271).

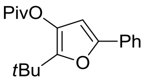

2-(*tert*-Butyl)-5-phenylfuran-3-yl pivalate (2d): Prepared following the **Procedure C** in 61% yield (63 mg, 0.21 mmol) from 104 mg (0.35 mmol) of **1d**. Colorless oil; TLC R_f 0.64 (pentane/Et_2O 10%); IR (neat) ν_{max} 1079, 1117, 1156, 1175, 1275, 1460, 1477, 1601, 1745, 2873, 2905, 2963, 2991, 3058 cm^{-1}; ^{1}H NMR (500 MHz, $CDCl_3$) δ 1.35 (s, 9H), 1.37 (s, 9H), 6.59 (s, 1H), 7.23 (t, J = 7.3 Hz, 1H), 7.35 (dd, J = 8.0, 7.3 Hz, 2H), 7.59 (d, J = 8.0 Hz, 2H); ^{13}C NMR (125.8 MHz, $CDCl_3$) δ 27.3, 29.0, 33.2, 39.1, 102.7, 123.4, 127.3, 128.7, 131.0, 134.0, 148.9, 149.1, 176.4; HRMS 323.161 ($C_{19}H_{24}O_3$ + Na calcd 323.162).

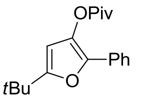

5-(*tert*-Butyl)-2-phenylfuran-3-yl pivalate (2d'): isolated as a mixture of **2d**/**2d'** using **Procedure C** from 104 mg (0.35 mmol) of **1d**. TLC R_f 0.71 (pentane/Et_2O 10%); ^{1}H NMR (400 MHz, $CDCl_3$) δ 1.32 (s, 9H), 1.39 (s, 9H), 6.24 (s, 1H), 7.21 (t, J = 7.3 Hz, 1H), 7.38 (dd, J = 8.0, 7.3 Hz, 2H), 7.68 (d, J = 8.0 Hz, 2H); ^{13}C NMR (125.8 MHz, $CDCl_3$) δ 27.4, 29.0, 33.1, 39.3, 101.2, 123.9, 126.7, 128.6, 130.3, 135.7, 138.4, 161.6, 176.0.

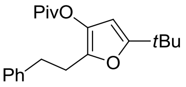

5-(*tert*-Butyl)-2-phenethylfuran-3-yl pivalate (2e): Prepared following the **Procedure C** in 78% yield (71 mg, 0.22 mmol) from 91 mg (0.28 mmol) of **1e**. Colorless oil; TLC R_f 0.52 (pentane/Et_2O 5%); IR (neat) ν_{max} 942, 1029, 1117, 1189, 1273, 1397, 1454, 1748, 2871, 2934, 2966, 3030, 3066, 3088 cm^{-1}; ^{1}H NMR (500 MHz, $CDCl_3$) δ 1.25 (s, 9H), 1.28 (s, 9H), 2.82 (t, J = 7.6 Hz, 2H), 2.92 (t, J = 7.6 Hz, 2H), 5.98 (s, 1H), 7.15 (d, J = 7.7 Hz, 2H), 7.20 (t, J = 7.4 Hz, 1H), 7.28 (dd, J = 7.7, 7.4 Hz, 2H); ^{13}C NMR (125.8 MHz, $CDCl_3$) δ 27.3, 27.5, 28.9, 32.9, 34.2, 39.1, 99.3, 126.1, 128.4, 128.5, 134.3, 140.3, 141.4, 160.6, 176.3; HRMS 351.191 ($C_{21}H_{28}O_3$ + Na calcd 351.193).

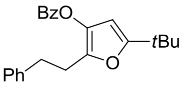

5-(*tert*-Butyl)-2-phenethylfuran-3-yl benzoate (2f): Prepared following the **Procedure C** in 81% yield (75 mg, 0.21 mmol) from 100 mg (0.29 mmol) of **1f**. Colorless oil; TLC R_f 0.45 (pentane/Et_2O 10%); IR (neat) ν_{max} 698, 1024, 1057, 1096, 1192, 1260, 1396, 1452, 1741, 2867, 2904, 2930, 2965, 3027 cm^{-1}; ^{1}H NMR (500 MHz, $CDCl_3$) δ 1.27 (s, 9H), 2.90 (t, J = 7.5 Hz, 2H), 2.96 (t, J = 7.5 Hz, 2H), 6.08 (s, 1H), 7.14−7.19 (m, 3H), 7.23−7.27 (m, 2H), 7.48 (dd, J = 8.3, 7.5 Hz, 2H), 7.62 (t, J = 7.5 Hz, 1H), 8.07 (d, J = 8.3 Hz, 2H); ^{13}C NMR (125.8 MHz, $CDCl_3$) δ 27.6, 29.0, 33.0, 34.1, 99.3, 126.1, 128.4, 128.6, 128.6, 129.4, 130.2, 133.6, 134.3, 140.8, 141.4, 160.8, 164.4; HRMS 371.165 ($C_{23}H_{24}O_3$ + Na calcd 371.163).

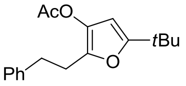

5-(*tert*-Butyl)-2-phenethylfuran-3-yl acetate (2g): Prepared following the **Procedure C** in 70% yield (71 mg, 0.48 mmol) from 101 mg (0.35 mmol) of **1g**. Colorless oil; TLC R_f 0.56 (pentane/Et_2O 10%); IR (neat) ν_{max} 1030, 1094, 1206, 1289, 1366, 1455, 1645, 1759, 2868, 2930, 2964, 3027, 3063, 3073 cm^{-1}; ^{1}H NMR (500 MHz, $CDCl_3$) δ 1.23 (s, 9H), 2.16 (s, 3H), 2.82 (t, J = 7.8 Hz, 2H), 2.91 (t, J = 7.8 Hz, 2H), 5.93 (s, 1H), 7.16 (d, J = 7.0 Hz, 2H), 7.19 (t, J = 7.5 Hz, 1H), 7.27 (dd, J = 7.5, 7.0 Hz, 2H); ^{13}C NMR (125.8 MHz, $CDCl_3$) δ 20.9, 27.4, 28.9, 32.9, 34.1, 99.2, 126.1, 128.4, 128.5, 134.1, 140.6, 141.4, 160.6, 168.8; HRMS 309.145 ($C_{18}H_{22}O_3$ + Na calcd 309.146).

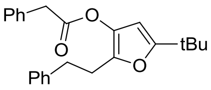

5-(*tert*-Butyl)-2-phenethylfuran-3-yl 2-phenyl acetate (2h): Prepared following the **Procedure C** in 75% yield (64 mg, 0.18 mmol) from 85 mg (0.23 mmol) of **1h**. Colorless oil; TLC R_f 0.42 (pentane/Et_2O 10%); IR (neat) ν_{max} 946, 1121, 1234, 1289, 1397, 1496, 1645, 1757, 2868, 2930, 2965, 3028, 3063, 3086 cm^{-1}; ^{1}H NMR (500 MHz, $CDCl_3$) δ 1.21 (s, 9H), 2.72 (t, J = 7.7 Hz, 2H), 2.82 (t, J =

7.7 Hz, 2H), 3.72 (s, 2H), 5.94 (s, 1H), 7.07 (d, *J* = 7.6 Hz, 2H), 7.18 (t, *J* = 7.3 Hz, 1H), 7.25 (t, *J* = 7.3 Hz, 2H), 7.28–7.36 (m, 5H); ^{13}C NMR (125.8 MHz, $CDCl_3$) δ 27.4, 28.9, 32.9, 34.1, 41.3, 99.1, 126.1, 127.5, 128.4, 128.5, 128.8, 129.4, 133.6, 134.2, 140.6, 141.4, 160.6, 169.3; HRMS 385.176 ($C_{24}H_{26}O_3$+Na calcd 385.177).

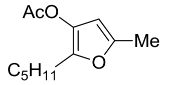

5-Methyl-2-pentylfuran-3-yl acetate (2i) [6]: Prepared following the **Procedure C** in 77% yield (77 mg, 0.37 mmol) from 100 mg (0.48 mmol) of **1i**. Colorless oil; TLC R_f 0.35 (pentane/Et_2O 5%); ^{1}H NMR (300 MHz, $CDCl_3$) δ 0.89 (t, *J* = 7.0 Hz, 3H), 1.25–1.36 (m, 4H), 1.54–1.62 (m, 2H), 2.22 (d, *J* = 1.0 Hz, 3H), 2.23 (s, 3H), 2.48 (t, *J* = 7.7 Hz, 2H), 5.94 (q, *J* = 0.9 Hz, 1H); ^{13}C NMR (125.8 MHz, $CDCl_3$) δ 14.1, 20.9, 22.5, 25.2, 27.6, 31.4, 102.7, 133.9, 142.3, 148.6, 169.1.

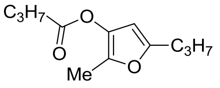

2-Methyl-5-propylfuran-3-yl butyrate (2j): Prepared following the **Procedure C** in 68% yield (71 mg, 0.33 mmol) from 104 mg (0.49 mmol) of **1j**. Colorless oil; TLC R_f 0.58 (pentane/Et_2O 10%); IR (neat) ν_{max} 943, 1149, 1240, 1372, 1456, 1652, 1761, 2874, 2933, 2962 cm^{-1}; ^{1}H NMR (400 MHz, $CDCl_3$) δ 0.95 (t, *J* = 7.5 Hz, 3H), 1.01 (t, *J* = 7.5 Hz, 3H), 1.62 (qt, *J* = 7.5, 7.5 Hz, 2H), 1.75 (qt, *J* = 7.5, 7.5 Hz, 2H), 2.14 (s, 3H), 2.47 (t, *J* = 7.5 Hz, 2H), 2.49 (t, *J* = 7.5 Hz, 2H), 5.96 (s, 1H); ^{13}C NMR (125.8 MHz, $CDCl_3$) δ 10.7, 13.8, 13.9, 18.6, 21.3, 30.6, 36.0, 102.1, 134.1, 137.9, 152.8, 171.6; HRMS 233.115 ($C_{12}H_{18}O_3$ + Na calcd 233.115).

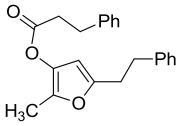

2-Methyl-5-phenethylfuran-3-yl 3-phenylpropanoate (2k): Prepared following the **Procedure C** in 74% yield (76 mg, 0.23 mmol) from 103 mg (0.31 mmol) of **1k**. Colorless oil; TLC R_f 0.51 (pentane/Et_2O 20%); IR (neat) ν_{max} 697, 747, 789, 933, 1075, 1135, 1235, 1371, 1453, 1495, 1651, 1758, 2860, 2921, 3027 cm^{-1}; ^{1}H NMR (400 MHz, $CDCl_3$) δ 2.09 (s, 3H), 2.83 (t, *J* = 7.8 Hz, 4H), 2.92 (t, *J* = 7.8 Hz, 2H), 3.04 (t, *J* = 7.8 Hz, 2H), 5.93 (s, 1H); 7.18−7.26 (m, 6H), 7.27−7.34 (m, 4H); ^{13}C NMR (125.8 MHz, $CDCl_3$) δ 10.7, 30.5, 31.1, 34.2, 35.7, 102.5, 126.2, 126.6, 128.5, 128.5, 128.5, 128.7, 134.0, 138.7, 140.2, 141.2, 151.8, 170.9; HRMS 357,145 ($C_{22}H_{22}O_3$ + Na calcd 357,146).

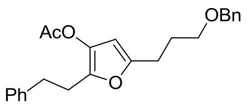

5-(3-(Benzyloxy)propyl)-2-phenethylfuran-3-yl acetate (2l): Prepared following the **Procedure C** in 65% yield (68 mg, 0.18 mmol) from 105 mg (0.28 mmol) of **1l**. Colorless oil; TLC R_f 0.43 (cyclohexane/EtOAc 20%); IR (neat) ν_{max} 578, 697, 734, 1071, 1099, 1206, 1367, 1453, 1495, 1759, 2856, 2925, 3027 cm^{-1}; ^{1}H NMR (400 MHz, $CDCl_3$) δ 1.84 (tt, *J* = 7.5, 6.3 Hz, 2H), 2.08 (s, 3H), 2.58 (t, *J* = 7.5 Hz, 2H), 2.72 (t, *J* = 7.7 Hz, 2H), 2.81 (t, *J* = 7.7 Hz, 2H), 3.43 (t, *J* = 6.3 Hz, 2H), 4.23 (s, 2H), 5.89 (s, 1H), 7.06–7.30 (m, 10H);^{13}C NMR (125.8 MHz, $CDCl_3$) δ 20.8, 25.3, 27.4, 28.1, 34.1, 69.4, 73.1, 102.4, 126.2, 127.7, 127.8, 128.5, 128.5, 134.2, 138.7, 141.1, 141.4, 152.4, 168.7; HRMS 402.175 ($C_{24}H_{26}O_4$ + Na calcd 402.176).
